# Changes in the outer nuclear layer and choroidal vascularity during the manifest and quiescent phases of acute central serous chorioretinopathy

**DOI:** 10.1038/s41598-024-67012-x

**Published:** 2024-07-11

**Authors:** Gyeongsoo Lim, Kyung Tae Kim, Dong Yoon Kim, Ju Byung Chae, Eoi Jong Seo

**Affiliations:** 1 Department of Ophthalmology, College of Medicine, Chungbuk National University Hospital, Chungbuk National University, 776, Sunhwan-1-Ro, Seowon-Gu, Cheongju, 28644 Korea; 2 Seoul Top Eye Center, Cheongju, Korea

**Keywords:** Central serous chorioretinopathy, Choroid, Optical coherence tomography, Photoreceptor cells, Subretinal fluid, Retinal diseases, Diagnostic markers

## Abstract

To investigate alteration of outer nuclear layer (ONL) and choroidal vascularity index (CVI) in different status of central serous chorioretinopathy (CSC). A retrospective review of 65 CSC eyes with subretinal fluid (manifest CSC) and 40 control eyes was conducted in a single tertiary university hospital. Differences in best-corrected visual acuity (BCVA), ONL, and CVI were compared. CVI was assessed both in the entire choroid (CVI-EC) and around the 1500 μm leakage area (CVI-1500). Measurements were repeated after the subretinal fluid resorption (quiescent CSC), and compared. CSC eyes showed worse BCVA, thinner ONL and greater CVI than controls. Quiescent CSC showed a recovery of ONL compared to the manifest CSC, along with the BCVA improvement. The resolution of the CSC revealed a decrease across all three choroidal areas (total, stromal and luminal), with a more pronounced reduction in the stromal than in the luminal choroidal area, leading to an increase in the CVI. This phenomenon was shown in both CVI-EC and CVI-1500. Conclusively, ONL thickness can be used as a quantitative biomarker for photoreceptor function in CSC. Increased CVI may reflect a disease activity. The stromal choroidal area is particularly sensitive in illustrating leakage from the choroidal vasculature.

## Introduction

Central serous chorioretinopathy (CSC) manifests through the occurrence of subretinal fluid (SRF) under the retina in macular region. This accumulation leads to a detachment of the neurosensory retina from the underlying tissue, which in turn can significantly disrupt central vision. This condition predominantly affects middle-aged individuals and is associated with a pathogenesis rooted in choroidal vascular hyperpermeability^1^. Various factors, including the use of systemic corticosteroids, type A personality traits, and other systemic stressors, have been identified as potential catalysts for CSC^2^. The underlying pathologic mechanism involves elevated hydrostatic pressure in the choroid, anomalous hyperpermeability of the choriocapillaris, and compromised barrier functionality of the retinal pigment epithelium (RPE), collectively contributing to macular fluid accumulation, photoreceptor damage, and visual disruption^3^.

Optical coherence tomography (OCT) has revolutionized the precise visualization of retinal and choroidal structures, offering unprecedented insights into the anatomical changes associated with CSC. In particular, outer nuclear layer (ONL) thickness has emerged as a crucial parameter in CSC assessment. By encompassing photoreceptor cell bodies, the ONL provides invaluable data about the health and functionality of these critical cells^4^. Its thickness has emerged as a potential quantitative biomarker reflecting the number of viable photoreceptors, with direct implications for central visual acuity in patients with CSC^5–7^. Concurrently, the choroidal vascularity index (CVI), a measure of the ratio between luminal and total choroidal area, serves as a quantitative biomarker for pathologic choroidal vascular dilation. The increase in CVI is due to dilated choroidal vessels, or increased numbers of choroidal vessels, or both^8^. CVI provides insights into both the pathogenesis of CSC and its prognosis, making it a cornerstone in understanding the overall trajectory^9–11^.

However, our understanding of retinal and choroidal changes, particularly regarding ONL thickness and CVI, during both the manifest and quiescent stages of CSC remains limited. While ONL thickness appears to increase following the resolution of SRF, these measurements are typically taken at a single point in the foveal center, and the precise underlying mechanism remains undisclosed^12,13^. Additionally, reports on CVI modifications following SRF resolution are inconsistent^14–16^. Therefore, this study aimed to compare the ONL and CVI between the manifest and quiescent status of CSC, as well as investigate their respective roles in the pathogenesis of the condition.

## Methods

We conducted a retrospective analysis of 65 eyes from 65 consecutive patients diagnosed with acute CSC who visited the Retina Clinic of Chungbuk National University Hospital between July 2020 and October 2022. Additionally, we included 40 eyes from age-matched normal controls. The Institutional Review Board of Chungbuk National University Hospital granted approval for this study, which was conducted in accordance with the ethical principles set forth in the Declaration of Helsinki.

The patient group included individuals diagnosed with acute CSC based on clinical history, spectral domain (SD) OCT (Spectralis optical coherence tomography, Heidelberg Engineering, Heidelberg, Germany), fluorescein angiography (FA, Heidelberg retinal angiography 2, Heidelberg Engineering, Heidelberg, Germany), and indocyanine green angiography (ICGA, Heidelberg retinal angiography 2, Heidelberg Engineering, Heidelberg, Germany). Specifically, eyes exhibiting SRF in the macular region on SD-OCT, pinpoint or smokestack leakage on FA, and choroidal vessel dilation with hyperpermeability on ICGA were defined as having CSC.

We included patients whose visual symptoms persisted for less than 3 months before visiting the clinic. Additionally, we selected only treatment-naïve patients who had not previously experienced visual symptoms or been treated with anti-vascular endothelial growth factor (VEGF), photodynamic therapy, or focal laser photocoagulation. Furthermore, we included only those patients whose SRF completely resolved within six months from baseline, regardless of whether they were treated (with anti-VEGF or focal laser) or observed. We defined the status of eyes with SRF as manifest CSC and the status with complete SRF absorption as quiescent CSC. Patients with recurrent or persistent SRF at the six-month follow-up were excluded from the study. In addition, patients with concomitant chronic CSC or pachychoroid neovasculopathy observed on ICGA, as well as those with other macular diseases such as diabetic retinopathy, retinal vascular occlusion, or choroiditis, and patients with high myopia (≥ − 5D), were also excluded.

For the control group, we selected 40 eyes of healthy patients without any observed retinal or choroidal diseases who visited the outpatient clinic with mild cataracts or presbyopia. This control group was age-matched to the CSC group to reduce age-related confounding effects.

**Figure 1:**
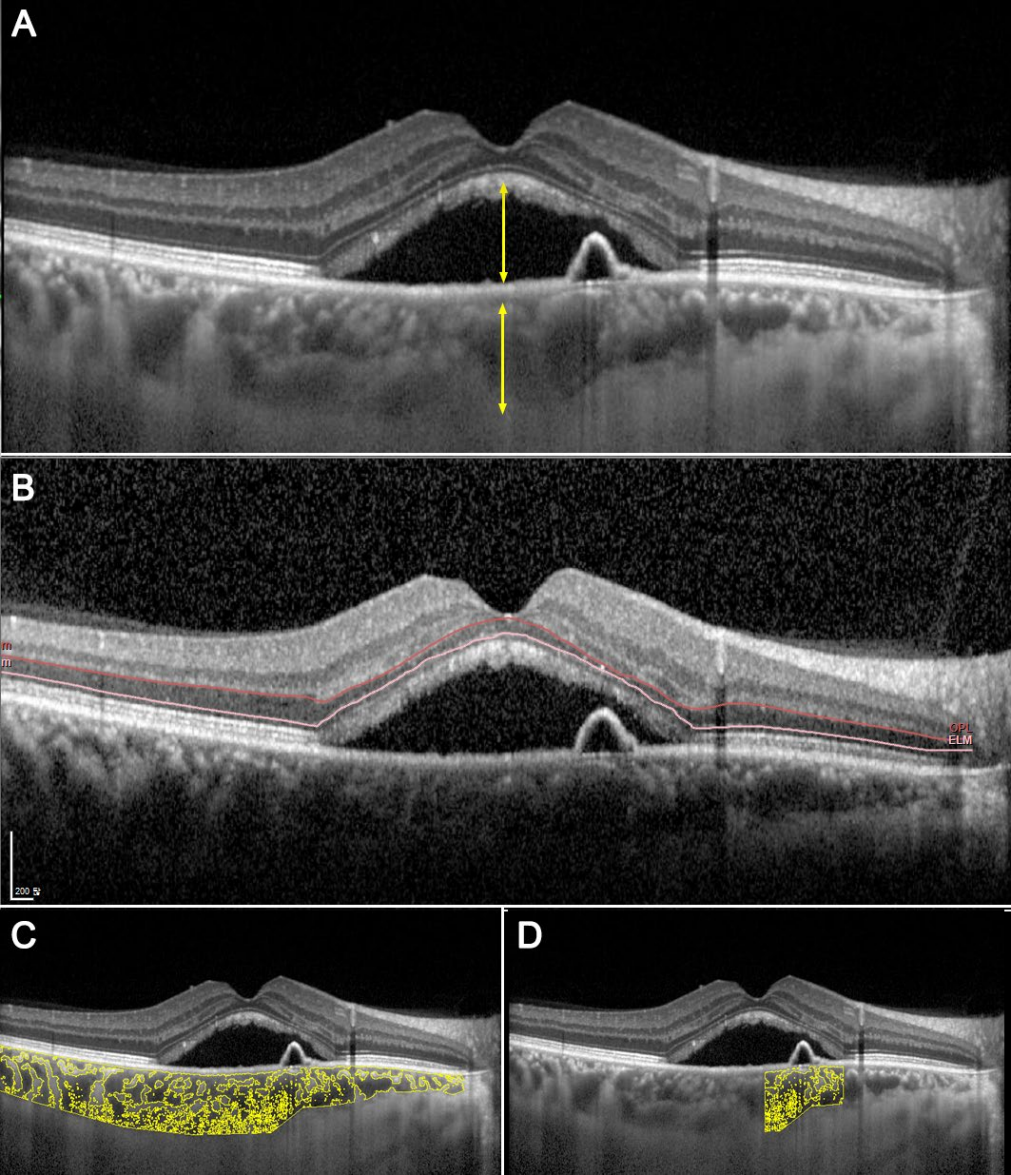
Demonstrations of measuring a subretinal fluid height and a subfoveal choroidal thickness (**A**), an average ONL thickness (**B**), a choroidal vascularity index (CVI) in the entire choroid (**C**), and a CVI around the leaking point (**D**). The outer plexiform layer and the external limiting membrane were demarcated and adjusted manually (**B**).

Data on age, sex, and spherical equivalents, as determined by automated refraction, were collected. All subjects underwent best-corrected visual acuity (BCVA) measurements, fundus examination, and SD-OCT imaging at baseline (manifest CSC) and 6 months later when the SRF completely resorbed (quiescent CSC). For the analysis, we used enhanced depth imaging (EDI) SD-OCT images, which provide horizontal cross-sections through the macula. The height of the foveal SRF was defined as the vertical length from the tip of the RPE to the detached ellipsoid zone at the fovea. Subfoveal choroidal thickness was measured as the distance from the outer surface of Bruch’s membrane to the choroid-sclera junction at the fovea (Fig. 1). The distance between the leakage point observed on the FA and the fovea was manually measured using software provided by the manufacturer. If there were multiple leaks, the nearest distance was used in the analysis.

ONL thickness was measured in three different ways to confirm the results. First, manual foveal ONL thickness measurement, which is the distance between the internal and external limiting membranes, was performed^5,12,13^. Second, the average thickness of the central 1 mm diameter circle was measured using the SD-OCT device with the embedded software provided by the manufacturer. Macular volume scans with 25 horizontal raster images were taken for the manifest and quiescent statuses. Each retinal layer was analyzed using automated segmentation. The outer plexiform layer and external limiting membrane were adjusted manually every 25 images of the scan because the automated segmentation was incorrect, especially in the presence of SRF (Fig. 1B). We then calculated the average thickness of the ONL within the central 1 mm diameter circle from the thickness map, both in the manifest and quiescent statuses. Third, the calculated ONL volume in the central 1 mm diameter circle based on the thickness map was collected and analyzed.

CVI in the entire choroid (CVI-EC) was measured using Image J software (Version 1.52; provided in the public domain by the National Institutes of Health, Bethesda, MD, USA; https://imagej.nih.gov/ij/) (Fig. 1C). The total choroidal area (TCA) was defined as the area of the choroid, extending vertically from the Bruch’s membrane to the choroid-sclera junction and horizontally from the optic disc to the edge of the image (approximately 7000 μm). To enhance measurement precision and reduce errors, we employed the average brightness of 3 to 4 choroidal vessels as the minimum brightness for image normalization. Subsequently, we converted the image to an 8-bit format and performed automatic binarization using the Niblack method with “Auto Local Threshold.” The darkened area was identified as the luminal choroidal area (LCA), while the white area represented the stromal choroidal area (SCA). The binarized image was then converted into an RGB (red, green, and blue) format. The dark area within the image was selected using “Color Threshold” and measured as the LCA. The CVI was calculated as the ratio of the LCA to the TCA. To ensure measurement reliability, two ophthalmologists (GL and EJS) conducted independent measurements, and the average of their results was used in the study. The intraclass correlation coefficients were 0.929 (95% confidence interval 0.865–0.962, *p* < 0.001) for the control group, 0.921 (95% confidence interval 0.870–0.952, *p* < 0.001) for manifest CSC, and 0.964 (95% confidence interval 0.941–0.978, *p* < 0.001) for quiescent CSC, respectively.

For a more detailed analysis, we also measured the CVI within a 1500 μm radius around the leakage point (CVI-1500), as depicted in Fig. 1D. This measurement was feasible in 46 out of the 65 eyes with CSC, depending on the quality of the OCT images. The measurement protocol was identical to that used for CVI-EC, with the only difference being the location, which was centered on the leakage point and covered a horizontal length of 1500 μm.

Statistical analysis was performed using SPSS software (Version 25.0; IBM Corp., Armonk, NY, USA). Continuous variables were expressed as mean ± standard deviation. All variables, except for BCVA, SRF height, subfoveal choroidal thickness, and ONL volume, passed the normality tests. The Student t-test was applied to variables that passed this test, while the Mann–Whitney test was used for those that did not. The parameters between manifest CSC and quiescent CSC were compared using paired t-tests. Linear regression analysis was performed to evaluate the relationship between BCVA and ONL thickness. Categorical variables are expressed as frequencies and percentages and were compared using the chi-square test. Differences were considered statistically significant when *P* values were less than 0.05.

## Results

A total of 65 eyes from 65 patients with acute CSC and 40 eyes from 40 age-matched healthy controls were analyzed. Initial BCVA was significantly poorer in the CSC group than in the control group (Table 1). No substantial differences were discernible between the two groups in terms of age or refractive error.

**Table 1:** Demographic features of the study population.

	CSC (manifest status)	Age-matched control	*p*-value
Total number (eyes)	65	40	
Sex (male/female)	51/14	25/15	0.076*
Age (years)	50.3 ± 8.2	51.7 ± 7.3	0.441†
BCVA (logMAR)	0.24 ± 0.24	0.03 ± 0.04	< 0.001‡
SRF height (µm)	207.3 ± 153.9	0.0 ± 0.0	< 0.001‡
Spherical equivalent (D)	− 0.21 ± 1.86	+ 0.08 ± 1.08	0.373†

*CSC* central serous chorioretinopathy; *BCVA* best-corrected visual acuity; *logMAR* logarithm of maximum angle resolution; *SRF* subretinal fluid; *D* diopter.

*p-value was calculated with chi-square analysis.

^†^*p*-values were calculated with Student *t*-test.

^‡^*p*-values were calculated with Mann–Whitney test.

**Figure 2:**
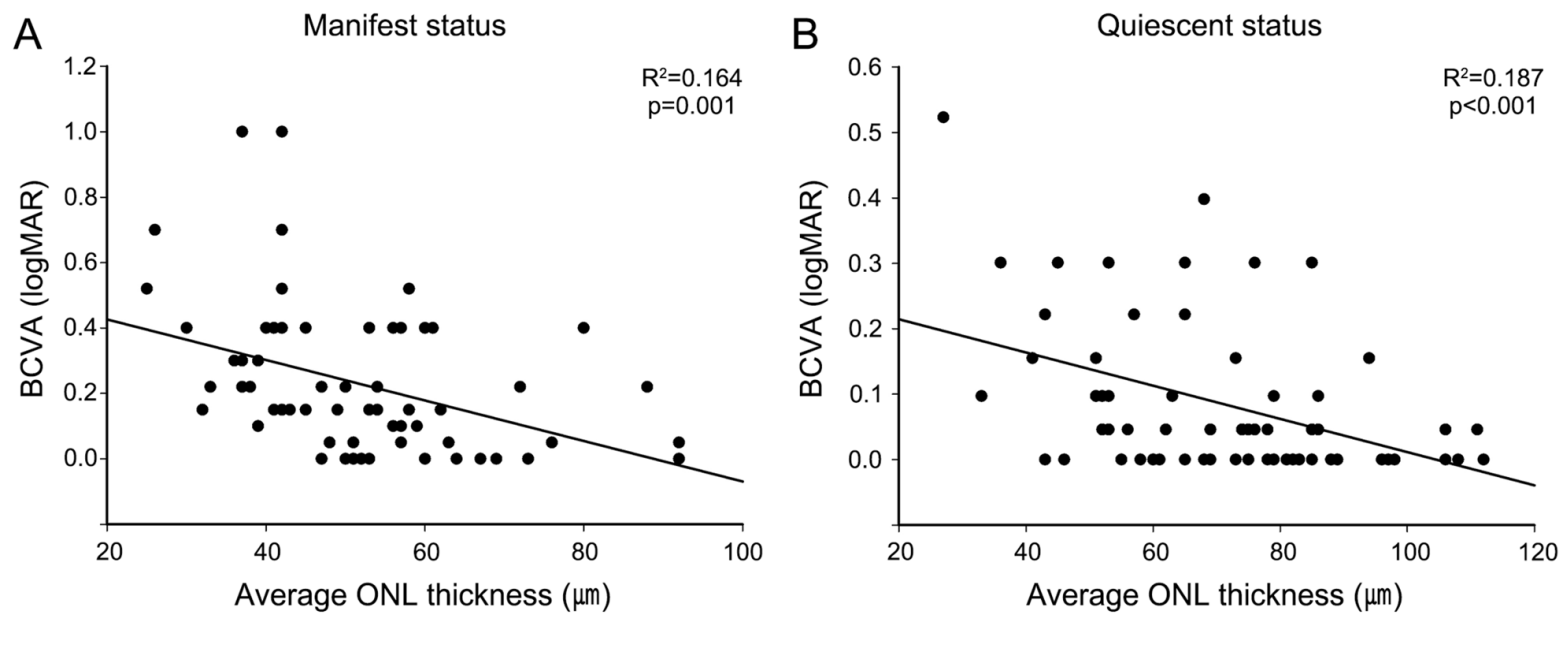
Linear regression analysis of the relationship between average outer nuclear layer (ONL) thickness and best-corrected visual acuity (BCVA) in the manifest (**A**) and quiescent (**B**) status of central serous chorioretinopathy (CSC). In both statuses, ONL thickness is negatively correlated with BCVA.

Changes in BCVA and ONL thickness, along with their relationship, were analyzed in CSC eyes for fluid resolution (Table 2). Decreased ONL thickness in manifest CSC was partially recovered with SRF resolution, and this alteration in ONL can be seen across all three measurement indices, namely foveal ONL thickness, average ONL thickness in the central 1 mm circle, and ONL volume in the central 1 mm circle. Concurrently, an enhancement in the BCVA has also been documented following SRF resorption. In linear regression analysis, a negative correlation was observed between average ONL thickness and BCVA in both the manifest (*p* = 0.001, R^2^ = 0.164) and quiescent (*p* < 0.001, R^2^ = 0.187) states of CSC (Fig. 2).

**Table 2:** Comparisons on outer nuclear layer in central serous chorioretinopathy and age-matched control eyes in three different measurements.

	Age-matched Control	CSC		*p*-value*	*p*-value†
		Manifest	Quiescent		
BCVA (logMAR)	0.03 ± 0.04	0.24 ± 0.24	0.09 ± 0.12	< 0.001	< 0.001
Foveal ONL thickness (µm)	106.6 ± 16.3	73.6 ± 21.4	85.5 ± 23.1	< 0.001	< 0.001
Average ONL thickness (µm)	90.0 ± 14.5	51.7 ± 14.7	70.0 ± 20.1	< 0.001	< 0.001
ONL volume (mm^3^)	0.07 ± 0.01	0.04 ± 0.01	0.05 ± 0.02	< 0.001	< 0.001

*CSC* central serous chorioretinopathy; *BCVA* best-corrected visual acuity; *logMAR* logarithm of the minimum angle of resolution; *ONL* outer nuclear layer.

Average ONL thickness and ONL volumes were calculated at the central 1 mm circle of the fovea.

*p-value represents a difference between the age-matched control and CSC (manifest) group, calculated using the Mann–Whitney test for BCVA and ONL volume, and the Student t-test for foveal and average ONL thickness.

^†^*p*-value represents a difference between CSC (manifest) and CSC (quiescent) group, calculated with paired *t*-test.

**Figure 3:**
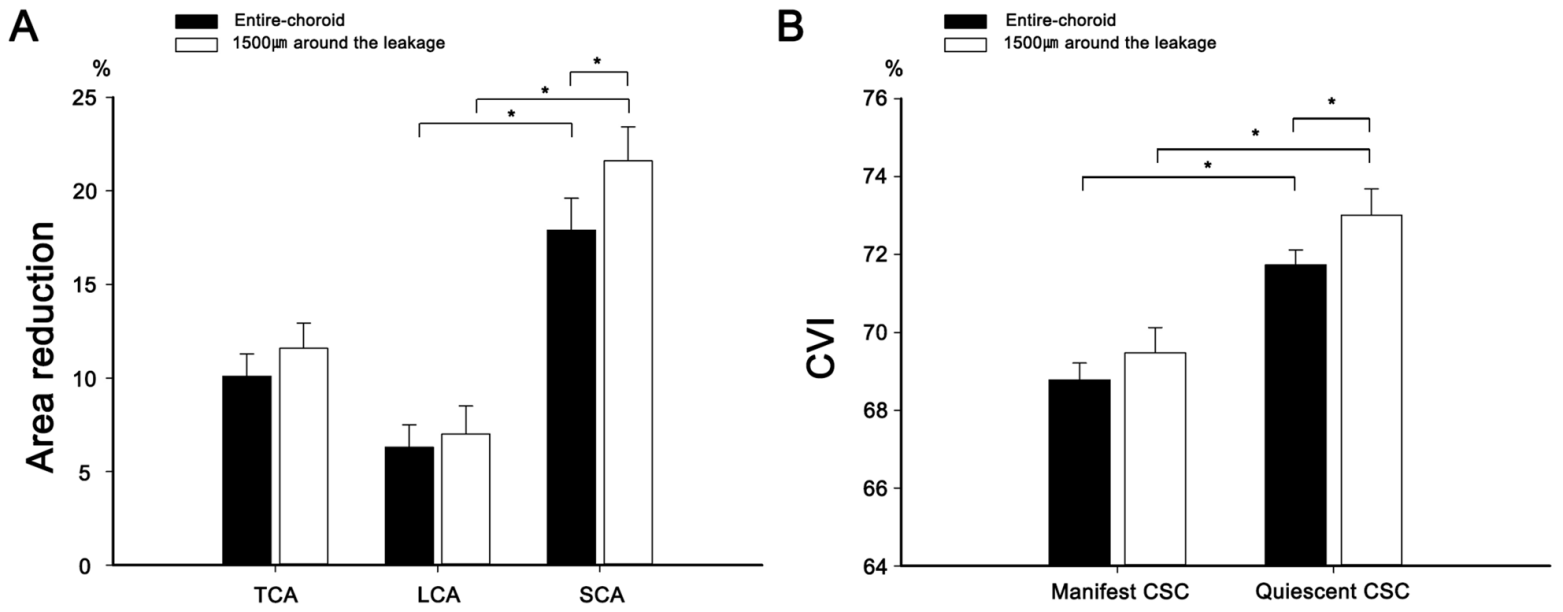
The area reductions in the total choroidal area (TCA), luminal choroidal area (LCA), and stromal choroidal area (SCA) following the resorption of subretinal fluid resorption, both in the entire choroid and 1500 μm around the leakage (**A**). Notably, the reduction in SCA was more prominent compared to that of LCA, resulting in an increase in choroidal vascularity index during the quiescent status compared to the manifest status in both measurements (**B**). Asterisks indicates that there are statistically significant differences (*p* < 0.05).

**Figure 4:**
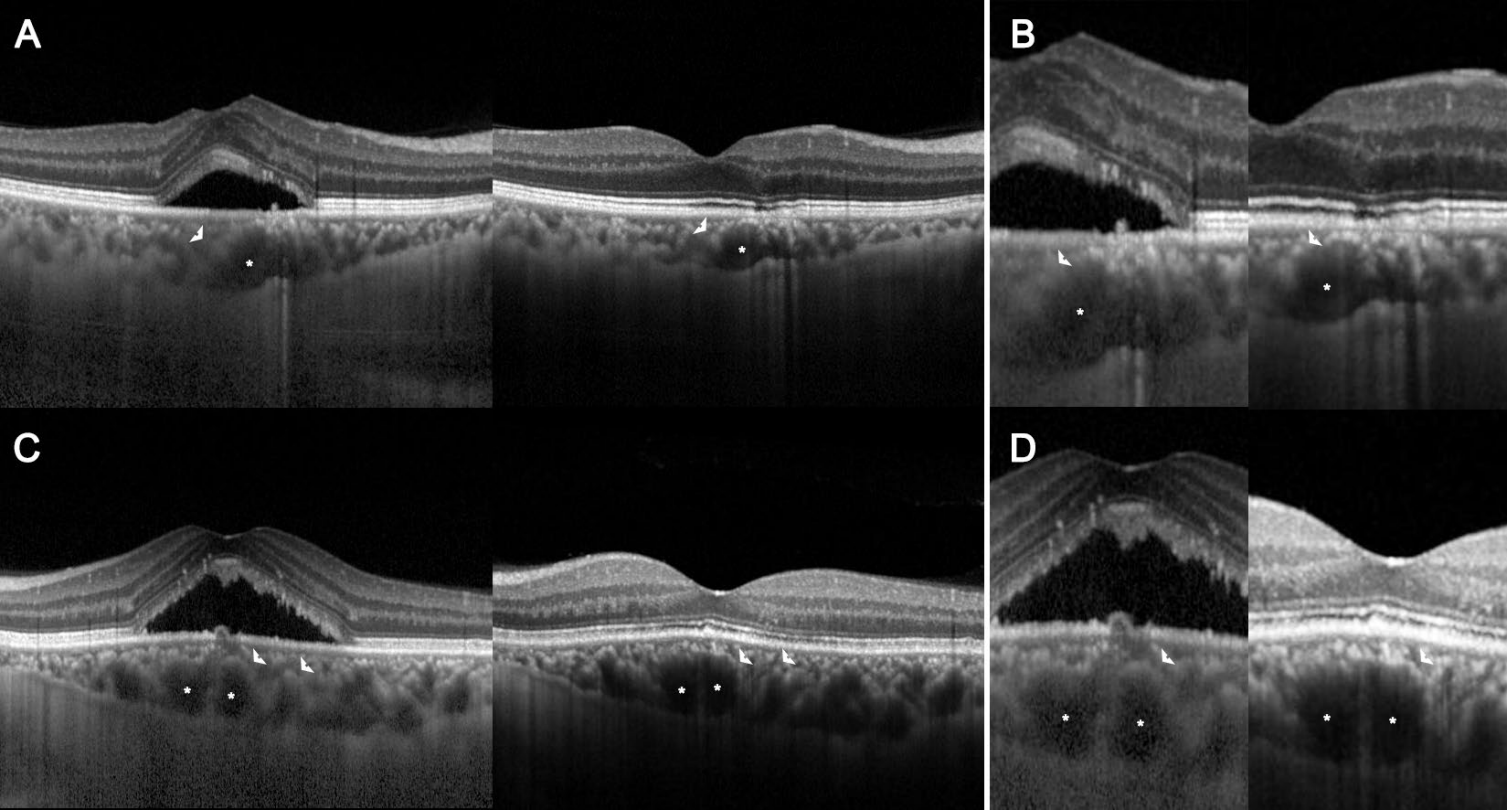
Representative optical coherence tomography images illustrating choroidal alteration in the manifest and quiescent status of central serous chorioretinopathy. The total choroidal area decreases as the subretinal fluid is absorbed, with the reduction in stromal choroidal area (SCA, arrowhead) exceeding that of the luminal choroidal area (LCA, asterisk). (**A**) and (**C**) demonstrate a change in the entire choroid while (**B**) and (**D**) highlight the change around the leaking point.

The subfoveal choroidal thickness in manifest CSC, which was greater than that in control eyes, decreased after SRF resolution (Table 3). Choroidal areas were measured in two independent manners: across the entirety of the choroidal area and within 1500 μm around the leakage point. In both measurement modalities, the TCA, LCA, and SCA were augmented in manifest CSC as opposed to age-matched controls. Subsequent resolution of the CSC revealed a decrease across all three choroidal areas, with a more pronounced reduction in the SCA than in the LCA, leading to an increase in the CVI (Fig. 3). Furthermore, the reduction in the SCA was greater around the leakage point (21.6 ± 12.3%) than across the entirety of the choroidal area (16.3 ± 12.1%, *p* = 0.028). Therefore, CVI in the quiescent CSC around the leakage point (73.01 ± 4.59%) was also greater than across the entirety of the choroidal area (71.73 ± 3.09%, *p* = 0.012). Representative images illustrating the changes in the choroidal area are shown in Fig. 4.

**Table 3:** Comparison between manifest and quiescent status of central serous chorioretinopathy.

	Age-matched control	CSC			*p*-value*	*p*-value†
		Manifest	Quiescent			
Sf ChThk (µm)	244.5 ± 44.3	363.9 ± 98.9		308.3 ± 96.6	< 0.001	< 0.001
*In the entire choroidal measurement*						
TCA (mm^2^)	3.948 ± 0.524	4.732 ± 1.449		4.225 ± 1.314	< 0.001	< 0.001
LCA (mm^2^)	2.616 ± 0.365	3.229 ± 0.896		3.011 ± 0.854	< 0.001	< 0.001
SCA (mm^2^)	1.331 ± 0.208	1.503 ± 0.579		1.215 ± 0.484	< 0.033	< 0.001
CVI-EC (%)	66.26 ± 2.74	68.78 ± 3.52		71.73 ± 3.09	< 0.001	< 0.001
*Around the leaking point measurement (1500 μm)*						
TCA (mm^2^)		1.410 ± 0.406		1.241 ± 0.350		< 0.001
LCA (mm^2^)		0.970 ± 0.246		0.897 ± 0.221		< 0.001
SCA (mm^2^)		0.440 ± 0.174		0.343 ± 0.145		< 0.001
CVI-1500 (%)		69.48 ± 4.41		73.01 ± 4.59		< 0.001

*CSC* central serous chorioretinopathy; *Sf ChThk* subfoveal choroidal thickness; *TCA* total choroidal area; *LCA* luminal choroidal area; *SCA* stromal choroidal area; *CVI-EC* choroidal vascularity index of the entire choroid; CVI-1500, choroidal vascularity index within 1,500 μm around the leakage point.

*p-value represents a difference between the age-matched control and CSC (manifest) group, calculated using the Mann–Whitney test for Sf ChThk and the Student *t*-test for all other variables.

^†^*p*-value represents a difference between the CSC (manifest) and CSC (quiescent) group, calculated with paired *t*-test.

## Discussion

We observed that patients with CSC exhibited a thicker choroid, a greater CVI, and a thinner ONL than those of the healthy controls. Remarkably, as the SRF resolved, partial recovery was noted in foveal ONL thickness, concurrent with visual improvement. Moreover, in the quiescent phase, the CVI further increased, primarily due to a more significant reduction in the SCA compared to the LCA. Notably, this reduction in the SCA was particularly prominent around the leaking area when compared to the entire choroid region.

The ONL thinning observed in CSC is in line with existing findings in chorioretinal disorders^6,17^. Similarly, pachychoroid epitheliopathy, characterized by thickened choroidal thickness and increased CVI but without SRF, also displayed reduced ONL thickness compared to normal controls^18^. Given that a typical ONL consists of 7–8 layers of photoreceptor cell bodies, its thickness serves as a potential indicator of viable photoreceptors and thus can be employed as a quantitative visual biomarker. The positive correlation between BCVA and ONL thickness in CSC, along with the discontinuity of the ellipsoid zone in eyes with thin ONL and poor BCVA, provides further support for ONL thickness as a visual biomarker^7^. Furthermore, ONL thickness was found to be reduced in eyes with longer symptom duration and poorer vision^6^.

The recovery of ONL thickness following the resolution of SRF in CSC was evident in our study, consistent with previous reports^12,13^. To enhance the reliability of ONL thickness change measurements, we employed automated assessments of averaged ONL thickness and volume within a central 1-mm diameter circle, with manual adjustments made in each OCT volume scan^12,13^. The ONL thickness recovery as the SRF resorption is considered to be related to the photoreceptor salvage, supported by numerous findings that the ONL thickness was correlated to BCVA^5–7,19,20^. Our research group has also recently reported ONL thickness recovery and its correlation with visual acuity improvement in the context of neovascular age-related macular degeneration treatment^21^. It is considered that the damaged photoreceptors without complete degeneration can be salvaged following the resolution of the SRF, resulting in ONL thickness recovery and improved visual acuity. Nevertheless, the use of ONL measurement as a visual biomarker should be interpreted with caution, as it may be influenced by the elasticity of retinal tissue^13^. Further studies involving serial observation of ONL thickness during SRF resorption can elucidate the mechanism behind these thickness changes.

Another novel finding of this study is the increase in the CVI with SRF resolution. CSC demonstrates thickened choroid with increased CVI^22^, or pachychoroid spectrum. Increased CVI was observed after SRF resorption of CSC, or even in the fellow eye of the CSC^23^. Therefore, the pachychoroid features and subsequent fluid dynamics from the choroid to the subretinal space are considered as a key pathogenic mechanism in CSC, PPE and associate disorders^24,25^. Investigation on the alteration of CVI before and after SRF resorption can provide detailed information of pathogenic choroidal characteristics and fluid dynamics in CSC.

However, previous reports on changes in CVI during SRF resolution have shown inconsistent results^14–16^. Agrawal et al. reported a decline in CVI with SRF resolution, possibly due to the choroidal blood flow decrease^14^. In contrast, others have reported that CVI remained unchanged between the manifest and quiescent states^15^. The inconsistency in the findings regarding CVI changes may be attributed to variations in measurement methods, such as some studies focusing on foveal CVI while others measured the entire choroid or the leaking points^14–16^. Various treatment methods, including photodynamic therapy, focal laser photocoagulation, and simple observation, can contribute to the different changes in CVI.

In our study, a higher CVI was observed in the quiescent status than in the manifest status. In the quiescent state, both SCA and LCA decreased. However, the extent of the decrease in SCA was greater than that in LCA, resulting in an eventual increase in the CVI. In manifest CSC, fluid moves from the dilated, leaky choroidal vessels to the adjacent stroma, leading to stromal oedema, an increase in choroidal hydrostatic pressure, and subsequent infiltration into the subretinal space. In the quiescent state, both stromal oedema and dilated choroidal vessels improved. The extent of stromal oedema improvement exceeded that of choroidal vessel shrinkage, leading to an increase in CVI. This indicates that SCA variability can sensitively represent disease activity, that is, vascular leakage into the choroidal stroma, rather than LCA variability. The great variability in SCA as a disease activity was in concordance with previous reports using ICGA, which described choroidal vessel dilation as well as diffuse leakage from the choriocapillaris into the stroma as features of the acute stage of the disease^26–28^.

To better understand the observed fluid dynamics, we conducted measurements of choroidal areas within a 1500 μm radius around the leakage point. This analysis revealed a similar phenomenon occurring around these leakage points. Moreover, we observed that the reduction in SCA was more pronounced at the leakage points compared to the entire choroid. This suggests a regional correlation between choroidal vascular leakage and RPE degeneration, as we identified the leakage points through pinpoint hyperfluorescence in FA, which indicates the breakdown of RPE tight junctions. This finding further emphasizes that choroidal stromal variability tends to outweigh choroidal vascular variability, whether assessed within a narrow region around the leakage point or across the entire macula.

In the clinical setting, monitoring choroidal characteristics and CVI usually provides useful information for both diagnosis and treatment of CSC. Increased choroidal thickness and CVI are associated with CSC, while age-related macular degeneration usually has a thin choroid^1,29^. The amount of choroidal reduction after SRF resolution can play an indicative role in predicting recurrence^30^. Decreased CVI may reflect changes in the choroidal vascular structure in eyes with CSC complicating choroidal neovascularization^10^. In this study, we also highlighted that monitoring CVI can provide insights into the disease activity, namely whether it is in a manifest or quiescent status. This information can help physicians in deciding on an individualized treatment modality.

Agrawal et al. reported results opposite to ours, with an increase in CVI following the resolution of the SRF in CSC^14^. This discrepancy could originate from either the measuring method or the treatment modality. Our study measured the CVI across the entire choroid and at leaking points, whereas they focused on the foveal center. We also analyzed CVI in a retrospective cohort, examining the same eyes at different disease statuses. In contrast, Agrawal et al. conducted a cross-sectional study, analyzing CVI in different eyes at various disease statuses^14^. Treatment modalities such as observation, photodynamic therapy, focal laser photocoagulation, and intravitreal anti-VEGF injection can also influence changes in choroidal characteristics. Recent observations have suggested that a decrease in choroidal thickness following anti-VEGF treatment in chronic CSC could serve as a potential biomarker for evaluating therapeutic responses^30,31^. However, in this study, there were no statistically significant differences in choroidal thickness or CVI changes observed with or without the use of anti-VEGF (see Table, Supplemental Digital Content 1, which represents a comparison of CSC eyes based on the treatment modalities). This observation can be attributed to the fact that our study included only acute CSC cases, which are seldom associated with choroidal neovascularization. For further clarification, future studies involving multiple time-point measurements that demonstrate linear alterations in CVI could strengthen the relationship between CVI and disease activity.

Our study presents several findings that contribute to a broader understanding of CSC and its associated factors. However, some of the inherent limitations of this study merit further consideration. First, the retrospective design of our study combined with the relatively limited number of participants may circumscribe the overarching generalizability of our conclusions. Second, the diversity of treatment modalities, including observation, focal laser, and anti-VEGF therapy, is a potential confounding factor. Although we clarified that there were no differences in choroidal thickness and CVI changes among the treatment modalities, there is still a possibility that the specific impact of each modality could account for our observed results. Furthermore, although notable ONL changes were observed, the underlying mechanisms remain unclear. We cannot conclusively determine whether these changes predominantly reflect the inherent viability of the photoreceptor or if they represent variations in the elasticity of the retinal tissue. Finally, our study lacked direct visualization of choroidal changes using indocyanine green angiography after fluid resolution. Although invasive, this tool may provide detailed insights into the changes in the choroidal vasculature before and after SRF resorption. Future studies integrating these methods may offer more comprehensive insights into disease processes and treatment responses.

In conclusion, we observed that patients with CSC exhibited distinct ocular changes, including increased choroidal thickness and CVI, and decreased ONL thickness, compared to healthy controls. As the SRF resolved, the foveal ONL thickness partially recovered, along with visual improvement, suggesting that the layer can play a role of visual biomarker. Regarding choroidal changes, increased CVI was noted in the quiescent state owing to a more significant reduction in the SCA than in the LCA, both in the entire macula and at the leaking point. This indicates that choroidal stromal variability represents disease activity more sensitively than choroidal vascular variability. Monitoring SCA, LCA, and CVI can provide information on disease activity and help develop a treatment plan for patients with acute CSC.

### Supplementary Information

Supplementary Table 1.

## Data Availability

The datasets generated during and/or analyzed during the current study are available from the corresponding author on reasonable request.
