# Supplementary material for: Changes in the outer nuclear layer and choroidal vascularity during the manifest and quiescent phases of acute central serous chorioretinopathy
Source: Sci Rep. 2024 Jul 11;14:16057. doi: 10.1038/s41598-024-67012-x (PMC11239832; doi:10.1038/s41598-024-67012-x)
Supplement: Supplementary file 1 — Supplementary Table 1. [file 41598_2024_67012_MOESM1_ESM.pdf]

Table S1. Comparison of central serous chorioretinopathy eyes treated with anti-VEGF and without anti-VEGF injection

	With anti-VEGF	Observation	p value
**Total number (eyes)**	30	31	
**Spherical equivalent (D)**	0.06 ± 2.17	-0.30 ± 1.05	0.410
**Visual acuity (logMAR)**			
Baseline (manifest status)	0.26 ± 0.22	0.20 ± 0.22	0.343
Resolution (quiescent status)	0.11 ± 0.14	0.07 ± 0.09	0.137
**ONL thickness (㎛)**			
Baseline (manifest status)	49.5 ± 14.7	54.0 ± 15.2	0.247
Resolution (quiescent status)	65.7 ± 18.3	75.8 ± 20.8	0.047
**Choroidal thickness (㎛)**			
Baseline (manifest status)	370.5 ± 109.7	357.5 ± 88.1	0.777
Resolution (quiescent status)	307.2 ± 97.7	306.0 ± 97.8	0.963
**CVI –EC (%)**			
Baseline (manifest status)	69.02 ± 2.87	68.73 ± 3.48	0.720
Resolution (quiescent status)	71.59 ± 3.08	71.62 ± 3.22	0.973
**CVI – 1500 (%)**			
Baseline (manifest status)	69.70 ± 3.03	68.92 ± 6.01	0.610
Resolution (quiescent status)	73.52 ± 3.81	72.02 ± 5.78	0.374

VEGF, vascular endothelial growth factor; logMAR, logarithm of maximum angle resolution; ONL, outer nuclear layer; CVI-EC, choroidal vascularity index of the entire choroid; CVI-1500, choroidal vascularity index of the leaking point 1500㎛ area

Focal laser photocoagulation and photodynamic therapy were excluded from this analysis due to the limited number of cases (4 and 0 eyes, respectively).
